# Galactose-Deficient IgA1-Specific Antibody Recognizes GalNAc-Modified Unique Epitope on Hinge Region of IgA1

**DOI:** 10.1089/mab.2018.0041

**Published:** 2018-12-28

**Authors:** Kohei Yamasaki, Hitoshi Suzuki, Junichi Yasutake, Yuji Yamazaki, Yusuke Suzuki

**Affiliations:** ^1^ Department of Nephrology, Juntendo University Faculty of Medicine, Tokyo, Japan.; ^2^ Nephrology R&D Unit, Kyowa Hakko Kirin Co., Ltd., Tokyo, Japan.

**Keywords:** ELISA, galactose-deficient IgA1 monoclonal antibody, epitope

## Abstract

Galactose-deficient IgA1 (Gd-IgA1) that exposes GalNAc or sialylated GalNAc has been shown to be associated with disease activity of IgA nephropathy (IgAN). In a previous report, we established an enzyme-linked immunosorbent assay that measures human Gd-IgA1 using a specific monoclonal antibody KM55 (KM55 mAb), and showed that patients with IgAN contain a higher level of serum Gd-IgA1 than other types of renal diseases. Recently, we also found that the KM55 mAb specifically recognized the glomerular-deposited Gd-IgA1 in renal biopsy. In this study, we aimed to analyze the epitope of KM55 mAb using synthesized peptides corresponding to the hinge region of IgA1 with GalNAc moiety on putative glycosylated Ser/Thr residues, which are Thr225, Thr228, Ser230, Ser232, and Thr236. Binding analysis to single GalNAc-modified hinge region peptide of IgA1 showed that Thr225 with GalNAc is required for recognition of KM55. PST(GalNAC)PP motif was required for KM55 mAb to recognize hinge region peptide of IgA1 which is shown by binding assay with deletion peptide. This result was confirmed by binding of KM55 mAb against peptide with GalNAc at Thr233, which resulted in containing another PST(GalNAC)PP motif. Taken together, we concluded that the epitope of Gd-IgA1-specific KM55 mAb is PST(GalNAc)PP motif.

## Introduction

IgA nephropathy (IgAN) is one of the most frequently diagnosed primary glomerulonephritis, characterized by deposition of IgA-based immunocomplex (IgA-IC) in the glomerular mesangial region.^(1)^ IgAN is common in Asian countries including Japan, where IgAN is the highest cause leading to end-stage renal disease followed by diabetic kidney disease. Currently, renin–angiotensin inhibitors and glucocorticoid steroids are widely used as basic therapy for IgAN, but due to their limited efficacy and side effects, basic study assessing the pathogenesis of IgAN is required for any new therapy.

There are two subclasses in human IgA: IgA1 and IgA2. IgA1 contains the hinge region that is located between the 223rd and 240th amino acids to connect its Fab and CH alpha constant region.^(2)^ Hinge region of IgA1 is characterized to have *O*-linked glycans on its Ser/Thr residues. Galactose-deficient IgA1 (Gd-IgA1), in which GalNAc or sialylated GalNAc residue is possibly exposed, has been found to be elevated in the circulation of patients with IgAN.^(3,4)^ Therefore, Gd-IgA1 is hypothesized to play a causative or progressive role in IgAN. To elucidate such pathogenic roles of Gd-IgA1, *Helix aspersa* aggulutinin (HAA) lectin has been widely used in basic and clinical studies, since HAA lectin is considered to exclusively bind to GalNAc residue on Gd-IgA1.^(5)^ However, it is known that HAA lectin includes some technical limitations due to lot-to-lot difference and instability.^(6)^ Therefore, a new research tool to detect Gd-IgA1 was desired.

In a previous report, we obtained Gd-IgA1-specific monoclonal antibody KM55 (KM55 mAb) by immunizing rats with a synthetic peptide corresponding to the hinge region of human IgA1 modified with GalNAc on putative *O*-glycosylated Ser/Thr residues along with IgAN patients pattern^(6)^ and consequently established an enzyme-linked immunosorbent assay (ELISA).^(7)^ Using this ELISA, we demonstrated that patients with IgAN contained a higher level of serum Gd-IgA1 than those with other types of renal diseases and healthy control, which was consistent with previous reports using HAA lectin assay.^(4,8,9)^ In addition, the KM55 mAb specifically recognizes the glomerular Gd-IgA1 in renal biopsy specimens.^(10)^ These results suggested that the KM55 mAb binds to specific forms of galactose deficiency in the hinge region of IgA1 abundantly observed both in circulation and in glomeruli of patients with IgAN. However, which forms of galactose deficiency in IgA1 KM55 recognizes have not been clarified. Therefore, in this study, we aimed to analyze the epitope of KM55 mAb by biochemical approaches.

## Materials and Methods

### Preparation of synthetic hinge region peptides of human IgA1 with GalNAc

All of the hinge region peptides of IgA1 used in this study are summarized in Table 1. All peptides were purchased from Sigma-Aldrich Japan (Tokyo, Japan). N terminus of all peptides was extended with two residues of Glycin, followed by conjugation with biotin through 10 polyethylene glycol. C terminus of all peptides is conjugated with amide. GalNAc-modified Ser/Thr residues were chemically synthesized and integrated into each peptide during the synthesis process.

**Table 1.** Sequences of IgA1 Hinge Region Peptides Used in Monoclonal Antibody KM55 Epitope Analysis

*Peptide number*	*Sequence*
	225 228 230 232 233 236
P1	P-S-T-P-P-T-P-S-P-S-T-P-P-T-P-S-P-S
P2	P-S-**T**-P-P-**T**-P-**S**-P-**S**-T-P-P-**T**-P-S-P-S
P3	P-S-**T**-P-P-T-P-S-P-S-T-P-P-T-P-S-P-S
P4	P-S-T-P-P-**T**-P-S-P-S-T-P-P-T-P-S-P-S
P5	P-S-T-P-P-T-P-**S**-P-S-T-P-P-T-P-S-P-S
P6	P-S-T-P-P-T-P-S-P-**S**-T-P-P-T-P-S-P-S
P7	P-S-T-P-P-T-P-S-P-S-T-P-P-**T**-P-S-P-S
P8	P-S-T-P-P-T-P-S-P-S-**T**-P-P-T-P-S-P-S
D1	P-S-**T**-P-P-**T**-P-**S**-P-**S**
D2	P-S-**T**-P-P-**T**-P-**S**-P
D3	P-S-**T**-P-P-**T**-P-**S**
D4	P-S-**T**-P-P-**T**-P
D5	P-S-**T**-P-P-**T**
D6	P-S-**T**-P-P
D7	P-S-**T**-P
D8	P-S-**T**

GalNAc residues were attached to specific amino acids underlined.

### Peptide binding assay

Each biotinylated peptide was diluted in phosphate-buffered saline (PBS) at 100 ng/mL and incubated on a streptavidin-coated 96-well plate (Roche Diagnostics, Basel, Switzerland) for 18 hours at 4°C, which was followed by blocking with 1% BSA/PBS (Nacalai Tesque, Kyoto, Japan) for 1 hour at room temperature. KM55 mAb was incubated on the peptide-immobilized plate for 2 hours at room temperature. After washing with 0.1% tween contained PBS (Wako Pure Chemical Industries Ltd., Osaka, Japan), 1/1000 diluted HRP-conjugated antirat IgG (Southern Biotec, AL) was incubated for 2 hours. After washing, the plate was colored by SIGMAFAST *O*-phenylenediamine solution (Sigma-Aldrich Japan) and the reaction was stopped by 0.5 mol/L sulfuric acid (Wako Pure Chemical Industries Ltd.). The absorbance at 492 nm of each well was measured. Rat IgG2b antibody (R&D systems, Inc., MN) was used as an isotype-matched control.

## Results

### GalNAc-modified Thr225 within hinge region of IgA1 is an essential residue for recognition of KM55 mAb

**FIG. 1.**
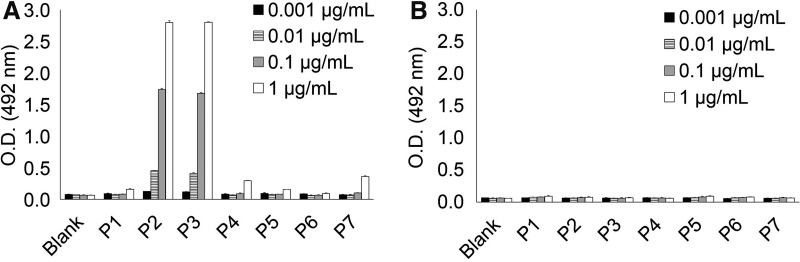
Binding analysis using hinge region peptides of IgA1 with various patterns of single GalNAc modification on each peptide (P1 to P7). Peptide sequence and GalNAc modification pattern are shown in Table 1. KM55 mAb showed robust binding to peptides P2 and P3, both of which possess GalNAc residue at Thr225, whereas KM55 mAb did not bind to other peptides (P1 and P4 to P7) **(A)**. In the same condition, isotype control antibody did not show binding to any of these peptides **(B)**. Error bar represents mean ± SD of triplicate assay. KM55 mAb, monoclonal antibody KM55; SD, standard deviation.

KM55 mAb was originally established by immunizing rats with the hinge region peptide of IgA1 with five GalNAc residues at Ser230, Ser232, Thr225, Thr228, and Thr236, where *O*-glycosylation was reported to be aberrant on IgA1 isolated from IgAN patient serum.^(11–13)^ To investigate whether recognition of KM55 mAb to this peptide can be determined by a single GalNAc-modified residue, we conducted binding assay using hinge region peptides of IgA1 with single GalNAc moiety at each putative glycosylated Ser/Thr residues (P3 to P7, Table 1) and peptide with multiple GalNAc at all of these Ser/Thr residues (P2, Table 1). As shown in Figure 1, KM55 mAb bound to peptides P2 and P3, whereas no robust binding was observed with peptides P4, P5, P6, and P7. These results indicate that GalNAc modification on a single amino acid residue is enough for KM55 mAb recognition, and GalNAc residue at Thr225, which is shared by peptides P2 and P3, is an essential residue for recognition of KM55 mAb.

### _223_PST(GalNAc)P_227_P is an essential motif for recognition of KM55 mAb

**FIG. 2.**
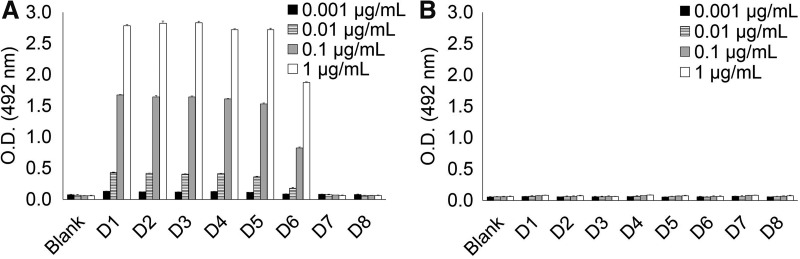
Binding analysis to deletion peptides of IgA1 hinge region. Sequence and modification of these peptides are shown in Table 1. Peptides D1 through D6 commonly contain _223_PST(GalNAc)P_227_P motif and peptides D7 and D8 lack _223_PST(GalNAc)P_227_P motif. KM55 mAb showed binding to peptides D1 through D6, whereas it did not bind to peptides D7 and D8 **(A)**. In the same condition, isotype control antibody did not show significant binding to these peptides **(B)**. Error bar represents mean ± SD of triplicate assay.

To elucidate which motif containing GalNAc-modified Thr225 *per se* is important for recognition of KM55 mAb, we synthesized deletion peptides with various lengths in amino acids after Thr225 (D1 to D8, Table 1). As shown in Figure 2, KM55 mAb showed binding to peptides D1 through D6, which share PST(GalNAc)PP motif. In contrast, binding against neither peptides D7 nor D8 that lack Pro226 and Pro227 was observed. These results indicate that Pro226 and Pro227 residues that are shared by peptides D1 and D6 are also required for KM55 mAb recognition, as well as GalNAc-modified Thr225. Therefore, _223_PST(GalNAc)P_227_P motif is possibly an essential motif for recognition of KM55 mAb.

### PST(GalNAc)PP is the KM55 mAb-recognizing motif regardless of its location

**FIG. 3.**
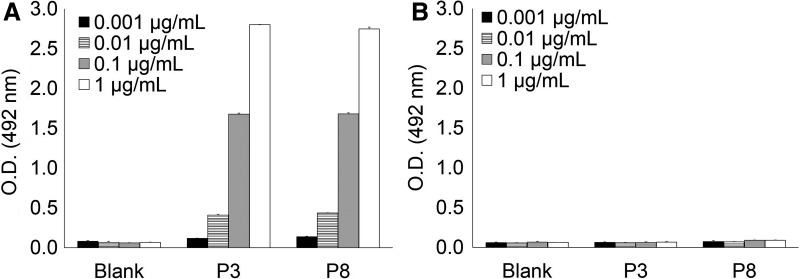
Binding analysis to peptide P8 with single GalNAc residue at Thr233, which resulted in containing another PST(GalNAc)PP motif at a different site from _223_PST(GalNAc)P_227_P (Table 1). KM55 mAb showed robust binding to peptide P8 **(A)**. In the same condition, isotype control antibody did not show significant binding to these peptides **(B)**. Error bar represents mean ± SD of triplicate assay.

To further confirm whether PST(GalNAc)PP motif *per se* in hinge region of IgA1 is the essential element for recognition of KM55 mAb, we synthesized peptide P8 containing single GalNAc modification at Thr233, which resulted in another PST(GalNAc)PP motif among hinge regions of IgA1 (Table 1). As shown in Figure 3, KM55 mAb bound to peptide P8 as it did to peptide P3. Taken together, the series of results indicate that KM55 mAb recognizes PST(GalNAc)PP motif, regardless of its position within hinge region of IgA1.

## Discussion

Our binding experiments using various types of hinge region peptides of IgA1 with single GalNAc-modified Ser/Thr residue indicated that single GalNAc modification at Thr225 among five Ser/Thr residues was enough for antigen recognition of KM55 mAb. Results from binding assay with deletion peptides indicated that PST(GalNAc)PP was the required motif for antigen recognition of KM55. Location of this motif within hinge region of IgA1 is unlikely to be important, because artificially created PST(GalNAc)PP motif at a different site on the hinge region of IgA1 also showed robust binding of KM55 mAb. Based on these results, we concluded that the epitope of KM55 mAb is the PST(GalNAc)PP motif. Considering that binding of KM55 mAb to peptide D6 that consists of a simple PST(GalNAc)PP motif was weaker than those to peptides D1 through D5, binding affinity of KM55 mAb might be affected by the existence of an amino acid on the bC-terminus side of PST(GalNAc)PP. Takahashi et al. reported that the galactose-deficient form of *O*-glycan at Thr233 is more frequently observed than that at Thr225 in serum IgA1 isolated from healthy control and IgA1 myeloma protein,^(13)^ which implies that serum Gd-IgA1 detected by KM55 ELISA predominantly contains P_231_ST(GalNAc)PP_235_ sequence rather than P_223_ST(GalNAc)PP_227_.

Although the galactose-deficient pattern of circulating IgA1 was already reported,^(14–16)^ that of glomerular-deposited IgA1 has not been clarified. We previously showed that Gd-IgA1 was deposited on the mesangial region of glomeruli specifically in patients with IgAN through immunofluorescent analysis using KM55 mAb.^(10)^ Taking our present study into consideration, glomerular-deposited Gd-IgA1 in patients with IgAN contains PST(GalNAc)PP motif in its hinge region. Further analysis will be required to elucidate which pattern of Gd-IgA1 is predominantly deposited to glomeruli with IgAN.

HAA lectin has been widely used as a Gd-IgA1 detection tool because of its specificity against GalNAc. In the previous study, we demonstrated that the serum level of Gd-IgA1 detected by KM55 mAb and HAA lectin is positively correlated and that the KM55 mAb partially inhibited binding of HAA lectin to Gd-IgA1 in a dose-dependent manner in competitive binding analysis,^(7)^ suggesting that both KM55 mAb and HAA lectin recognize the same site on Gd-IgA1. However, Gomes et al. revealed by binding assay combined with surface plasmon resonance spectroscopy that HAA lectin bound predominantly to GalNAc at Thr228, Ser230, and Ser232,^(5)^ which are different from the putative binding site of KM55 mAb elucidated in this study. Therefore, it is still not clear whether KM55 mAb and HAA lectin share a single binding site within hinge region of Gd-IgA1. Further research will be needed to elucidate the relationship between these two tools.

Our study has several limitations. (1) Galactose-deficient *O-*glycan in IgAN patients also includes sialic acid modification directly onto GalNAc residue. However, galactose deficiency was mimicked by simply adding GalNAc residues to naked Ser/Thr amino acids in this study. Therefore, this study could not evaluate involvement of sialic acid in recognition of KM55 mAb. (2) All experiments were conducted using synthesized peptides with GalNAc residues that do not always take structure identical to the endogenous hinge region of Gd-IgA1. Moreover, glycosylation itself at specific Ser/Thr residues can alter the angles of adjacent chains of amino acid, which can influence the recognition of KM55 mAb.^(17)^ (3) Actual pathophysiological *O-*glycosylation pattern is considered to be heterogeneous; therefore, various types of Gd-IgA1 molecules can be found even in the circulation of a single patient, not to mention, among individuals who have different clinical episodes. To identify what type of *O*-glycosylation KM55 mAb recognizes an endogenous Gd-IgA1 in patients with IgAN, further analysis will be needed, such as physicochemical analysis of complex between patient-derived Gd-IgA1 and KM55 mAb.

In conclusion, we could successfully identify the epitope of KM55 mAb in the amino acid level, including glycan modification by peptide binding assays, which impacts on potential importance of Gd-IgA1 containing the identified epitope PST(GalNAc)PP. This study will be helpful to appropriately interpret the study results obtained by KM55 mAb, which leads to not only a deep understanding of IgAN pathogenesis but also various clinical applications of KM55 mAb for diagnosis and prognosis of IgAN disease activity.
